# Tolcapone in Obsessive Compulsive disorder: A Randomized Double-Blind Placebo-Controlled Crossover Trial

**DOI:** 10.1097/YIC.0000000000000368

**Published:** 2021-09-01

**Authors:** Jon E. Grant, Roxanne Hook, Stephanie Valle, Eve Chesivoir, Samuel R. Chamberlain

**Affiliations:** 1 University of Chicago, Department of Psychiatry and Behavioral Neuroscience, Chicago, IL USA; 2 Department of Psychiatry, University of Cambridge, UK; 3 Department of Psychiatry, Faculty of Medicine, University of Southampton; and Southern Health NHS Foundation Trust, Southampton, UK

**Keywords:** obsessive compulsive disorder, treatment, pharmacology, COMT inhibitor, tolcapone

## Abstract

**Objective:**

Despite the availability of evidence-based treatments for obsessive-compulsive disorder (OCD), not all patients experience sufficient benefit or are able to tolerate them. Tolcapone is a catechol-o-methyl-transferase (COMT) enzyme inhibitor that augments cortical dopaminergic transmission. Conduct a proof of concept study to examine whether a COMT inhibitor would reduce OCD symptoms to a greater extent than placebo.

**Methods:**

We conducted a randomized, placebo-controlled, double-blind cross-over trial in adults with OCD (N=20). Participants were assessed at baseline, after 2 weeks of tolcapone, and again after 2 weeks of placebo on measures of OCD symptom severity and psychosocial functioning. There was a one-week washout period between the two-week treatment phases.

**Results:**

Two weeks of tolcapone was associated with significant improvement in OCD versus two weeks of placebo (t=2.194, p=0.0409). The mean percentage decreases in the total YBOCS scores for the entire sample over the corresponding two-week periods were 16.4% for tolcapone and 3.6% for placebo.

**Conclusions:**

These data indicate that brain penetrant COMT inhibitors merit further investigation as a candidate new treatment for OCD.

## Background

Obsessive-Compulsive Disorder (OCD) is characterized by repetitive intrusive thoughts (obsessions) and/or repetitive rituals undertaken in response to those thoughts, or according to rigid rules (APA, 2013). OCD is a common psychiatric health disorder (prevalence rate of approximately 2%) and is often accompanied by increased anxiety, depression, and other psychosocial dysfunction (Grant, 2014). Current first-line treatments include cognitive behavioral therapy using exposure response prevention and/or serotonin reuptake inhibitors (SRIs) (Skapinakis et al., 2016). While helpful for many patients, not everyone can tolerate these interventions (e.g. SRIs often have intolerable sexual side effects), and up to 35% of people do not experience adequate symptom relief from them (Grant, 2014).

Tolcapone, a catechol-O-methyl-transferase (COMT) inhibitor, is available in some geographical jurisdictions (including the UK and USA) as an add-on agent for the specialist management of Parkinson’s Disease. The enzyme COMT serves to break down free dopamine in the prefrontal cortex; by blocking this enzyme, tolcapone enhances dopamine signaling in the cortex. In the frontal cortex, optimal dopamine modulation of prefrontal cortical networks appears to be necessary for a variety of cognitive functions, including planning, inhibition, attention, and response flexibility (Tunbridge et al., 2004; Robbins et al., 2009; Cools et al., 2011). Importantly, dysfunction of many of these cognitive domains has been implicated in OCD (Chamberlain et al., 2005; Fullana et al., 2020). Tolcapone has been found to modulate prefrontal cortex function in other settings, including when given over relatively short treatment durations (Ashare et al., 2013). Previously, open-label treatment with tolcapone was associated with symptomatic improvements in gambling disorder, which correlated with normalizing effects on brain activation during an executive planning task (Grant et al., 2013). Controlled studies have also reported some cognitive enhancing effects of tolcapone in healthy controls e.g. (Apud et al., 2007). Tolcapone may therefore offer promise for the treatment of OCD. Furthermore, our clinical experience suggests that when people are treated with tolcapone, they often report benefit within one to two weeks after starting the medication.

The aim of the current study was to examine the efficacy of two-week treatment with tolcapone in adults with OCD. This was a randomized, double-blind, placebo-controlled crossover design with a one-week washout between treatment periods. We hypothesized that tolcapone would significantly improve symptoms of OCD compared to placebo.

## Methods

Participants were recruited via advertisements and referrals, and were adults, aged 18-65 with a primary current Diagnostic and Statistical Manual Version 5 (DSM-5) diagnosis of OCD. All participants were recruited at a single site. Inclusion criteria included a current diagnosis of OCD with a minimum YBOCS severity score of 18. Participants were included if they were currently taking psychotropic medications as long as the dose had been stable for at least 3 months (only 5 were taking medication; see Results). Exclusion criteria were: 1) Unstable medical illness, including liver disease, as determined by the investigator and liver function test; 2) History of seizures; 3) Clinically significant suicidality (defined by the Columbia Suicide Severity Rating Scale); 4) Baseline score of ≥17 on the Hamilton Depression Rating Scale (17-item HDRS); 5) Lifetime history of bipolar disorder type I or II, schizophrenia, autism, any psychotic disorder, or any substance use disorder; 6) Initiation of psychotherapy or behavior therapy within 3 months prior to study baseline; 7) Previous treatment with tolcapone; 8) Any history of psychiatric hospitalization in the past year; and 9) Currently pregnant (confirmed by urine pregnancy test).

Diagnosis was made using the clinician-administered *Structured Clinical Interview for DSM-5* (First et al., 2015), and the *Mini-International Neuropsychiatric Interview 7.0* (Sheehan et al., 1998) was used to screen for co-occurring psychiatric disorders. OCD symptom severity at baseline and follow-up visits were examined using the *Yale Brown Obsessive Compulsive Scale (YBOCS)* (Goodman et al., 1989) (the YBOCS was administered by the first author who has been trained in the use of the YBOCS and has twenty years of experience using the instrument). Change in total YBOCS scores constituted the a priori primary outcome measure. We also assessed secondary outcome measures of psychosocial impairment using the *Sheehan Disability Scale* (SDS) (Sheehan, 1983); anxiety using the *Hamilton Anxiety Rating Scale (HAM-A)* (Hamilton, 1959) and depression with the *Hamilton Depression Rating Scale (HAM-D)* (Hamilton, 1960).

This was a fully counter-balanced, double-blind, placebo-controlled cross-over design in which participants received EITHER tolcapone 100mg twice daily for two weeks, followed by one-week washout, followed by placebo twice daily for two weeks; OR placebo twice daily for two-weeks, followed by one-week washout, followed by tolcapone 100mg twice daily for two weeks. The order of treatment was randomized. The washout period was intended top prevent possible carryover effects that might compromises the assessments and conclusions. The study had low risk of bias using the Cochrane criteria (see Risk of Bias Assessment Table, Supplemental Digital Content 1). Because tolcapone at these lower doses has few side effects, a cross-over design was considered as side effects should not jeopardize the blinding.

Participants were recruited in the study from the 19^th^ of March 2019 until the 16^th^ of September 2020. The authors assert that all procedures contributing to this work comply with the ethical standards of the relevant national and institutional committees on human experimentation and with the Helsinki Declaration of 1975, as revised in 2008. All procedures involving human subjects/patients were approved by the University of Chicago Institutional Review Board. After a comprehensive explanation of study procedures and an opportunity to ask any questions, all participants provided written informed consent. Participants were informed that tolcapone is not FDA-approved for the treatment of OCD and is therefore investigational. Participants were compensated 100 USD for time and travel associated with the four visits.

All efficacy and safety assessments were performed at each visit. Subjects who were not compliant with their use of study medication (i.e. failing to take medication for three or more consecutive days) were discontinued from the study.

An external pharmacy independent of the research team conducted the sequence generation using randomized lists. Medication was also assigned by the external pharmacy independent of the research team. All medication and placebo were of identical appearance, and were supplied as such to the study team and participants. All participants and study personnel were fully blinded. Outcome assessment was fully blinded.

### Safety Assessments

Safety and tolerability were assessed using spontaneously reported adverse events data, Columbia–Suicide Severity Rating Scale (C-SSRS) (Posner et al., 2011), vital signs, and by evaluating premature termination. Safety assessments (C-SSRS, sitting blood pressure, heart rate, adverse effects, and concomitant medications) were documented at each visit. Assessment of side effects was done at each visit. Adverse events were coded by system organ class and preferred term using the Medical Dictionary for Regulatory Activities (Med-DRA) Version 11.1. Liver function tests were performed at baseline and then at study endpoint until in-person office visits were not possible given the COVID19 pandemic. Due to the pandemic of COVID19, study participants were allowed to perform their baseline and follow-up visits online using encrypted videoconferencing with the clinician, instead of in person visits. Blood samples for assessment of liver function, therefore, were at the discretion of the study investigator, and where considered medically necessary, the participant had them drawn locally if possible and submitted to the study team.

### Data Analysis

The analysis followed Intent-To-Treat principles, with Last-Observation-Carried-Forward for dropouts and missing data. The analytic approach was determined prior to data unlock. Changes in YBOCS total scores for placebo treatment and tolcapone treatment were compared using paired sample t-tests, which constituted the primary analysis. This method was appropriate because the design was fully counter-balanced and randomized. Changes in total depression and anxiety symptom scores, and disability, were examined, again using paired t-tests, as secondary analyses. Statistical significance was defined as p<0.05 two-tailed.

## Results

A total of 28 participants signed informed consents and were enrolled. Of those, eight individuals chose not to proceed with the baseline assessment or were deemed ineligible due to exclusion criteria and were not included for analysis (see CONSORT Diagram, Supplemental Digital Content 1). Of the remaining 20 subjects, their demographic and clinical variables are presented in Table 1.

The mean duration of OCD illness was 18.3 (± 12.6) years. Of the 20 participants, 16 (80%) reported some previous treatment for OCD (either psychotherapy or medication). Of the 20 participants, 5 (25%) were taking other psychotropic medications prescribed specifically for the person’s OCD: two were taking sertraline (both at 100mg/d), two were taking fluoxetine (one on 40mg/d and one on 60mg/d), and one was prescribed venlafaxine (150mg/d). The current doses of medications had been stable for at least three months for each participant before enrolling in the current study and remained unchanged for the duration of the study. No participant was currently undergoing psychotherapy.

Of the 20 subjects, 9 (45%) had current psychiatric comorbidities based on the MINI. Five met criteria for major depressive disorder, two for panic disorder with agoraphobia; two met criteria for social anxiety disorder, two for post-traumatic stress disorder, two for generalized anxiety disorder, two for at least one substance use disorder, and one for antisocial personality disorder.

**Figure 1:**
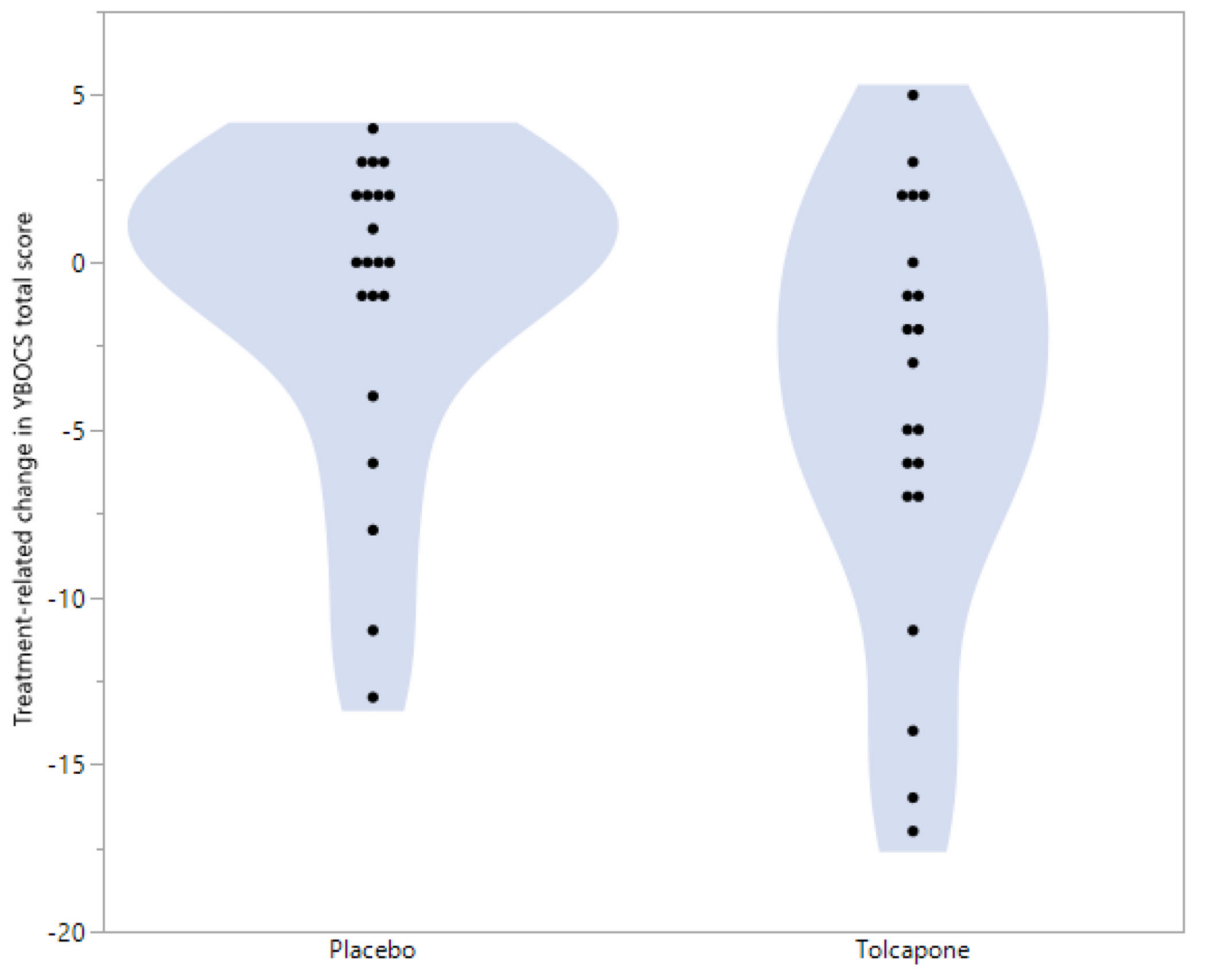
Violin plots for the change in YBOCS under each treatment condition.

When taking tolcapone, participants experienced a mean decrease in their total YBOCS scores of -4.24 (SD= 6.20), which differed significantly from the change observed under placebo treatment of -1.10 (SD= 4.71) (t=2.194, df=19, p=0.0409). Figure 1 shows violin plots for the change in YBOCS under each treatment condition. The mean percentage decreases in the total YBOCS scores for the entire sample over the corresponding two-week periods were 16.4% for tolcapone and 3.6% for placebo.

HAM-A scores changed by -1.90 (SD= 4.39) with active treatment and by -1.60 (SD= 4.07) with placebo; and HAM-D scores changed by -1.65 (3.67) with active treatment and by - 2.00 (4.42) with placebo. The changes between the treatment conditions were non-significant for these two measures (t=0.2037, df=19, p=0.841; and t=0.292, df=19, p=0.774). SDS total scores changed by -2.68 (SD =4.41) under active treatment and by -1.97 (SD= 6.62) under placebo conditions. The change between the treatment conditions on SDS was not significant (t=0.503, df=18, p=0.693). One person’s SDS data were missing at all time points (no data to carry forward).

Three adverse events occurred during active treatment, and three adverse events occurred during placebo treatment. All were of minor severity. Reported adverse events during tolcapone treatment were as follows: Mild headache, one patient; increased frequency of bowel movements, one patient; and blood in stool, one patient (this was followed up and found to be haemorrhoids). Reported adverse events during placebo treatment were as follows (each experienced by one patient per adverse event): Less frequent bowel movements, fatigue, muscle aching, and joint stiffness; and sleep problems. No suicidality was detected during the study at any time point on the C-SSRS.

## Discussion

This randomized, placebo-controlled cross-over trial indicated that two-week treatment using tolcapone (100mg twice daily) was associated with significant symptomatic improvement in OCD as compared to two-week administration of placebo. The likely mechanism is that tolcapone enhanced cortical dopamine transmission and therefore top-down executive control over habitual patterns of behavior. The finding of significant separation from placebo after two weeks may suggest that longer studies using tolcapone for OCD may be worthwhile to see if even greater benefit is possible. This finding of benefit after two weeks of medication is in fact not new to OCD. Early research on fluvoxamine found that although full response to medication took approximat5ely 8 weeks, the medication actually demonstrated statistically significant improvement compared to placebo after only two weeks of administration (Goodman et al., 1989). The percentage decrease in YBOCS total scores did not meet the threshold for “responder” generally defined by clinical trials, but the change in YBOCS in a two-week exposure was expected to be small and therefore it would be inappropriate to expect measures such as responder status to be relevant in this study. This small study was intended merely to see if tolcapone produced a signal and therefore would merit further study.

Limitations of this study include that the study design (including the extremely short duration of treatment) and the nature of the sample (primarily of individuals with relatively mild illness) precluded more complex statistical modelling. Additionally, a major confound in the study is the fact that some patients were being treated with SRIs and some were not. Although numerically those on SRIs do not appear to have had a better or worse response to tolcapone based on change in total YBOCS scores, this variable represents a confounder in our anlaysis.

These data indicate that tolcapone, a brain penetrant COMT inhibitor, merits further evaluation as candidate treatment for OCD, in clinical trials of greater sample size and duration than herein. The study may also raise questions about COMT inhibitors as a class of potential medications as they have a distinct neurochemical mode of action versus existing pharmacotherapies for OCD (e.g. SRIs), and may offer advantages in terms of enhancing top-down executive control (Tunbridge et al., 2004; Apud et al., 2007).

## Supplementary Material

Consort Diagram

## Figures and Tables

**Table 1:** Baseline demographics

	Mean (SD) / Percentage (N)	Range
**Age**	36.9 (12.73)	19-61
**Gender (% female)**	40% (8)	-
**Ethnicity**	-	-
**Caucasian**	60% (12)	-
**African American**	20% (4)	-
**Latino/Hispanic**	5% (1)	-
**Other**	10% (2)	-
**Missing data**	5% (1)	-
**Medication at baseline**	-	-
**Yes**	30% (6)	-
**No**	25% (5)	-
**Missing**	45% (9)	-
**Comorbidities**	-	-
**Yes**	45% (9)	-
**No**	55% (11)	-
**Education**	-	-
**High school graduate**	5% (1)	-
**Some college**	25% (5)	-
**College graduate**	40% (8)	-
**College +**	30% (6)	-
**YBOCS Total**	21.4 (4.06)	18-29
**HAM-A**	6.55 (5.84)	0-16
**HAM-D**	6.50 (5.80)	0-20

Comorbidities (N): Major depressive episode (5), panic disorder (2), agoraphobia (2), social phobia (2), post-traumatic stress disorder (2), substance dependence/abuse (2), generalized anxiety disorder (2), antisocial personality disorder (1). All comorbidities refer to current disorders. YBOCS: Yale-Brown Obsessive-Compulsive Scale; HAM-A: Hamilton Anxiety Rating Scale; HAM-D: Hamilton Depression Rating Scale.
