# Supplementary material for: Tolcapone in Obsessive Compulsive disorder: A Randomized Double-Blind Placebo-Controlled Crossover Trial
Source: Int Clin Psychopharmacol. Author manuscript; Available in PMC 2021 Sep 1. (PMC7611531; doi:10.1097/YIC.0000000000000368)
Supplement: Consort Diagram [file EMS123355-supplement-Consort_Diagram.docx]

**CONSORT DIAGRAM. Subject Flow Diagram for Tolcapone versus Placebo in the Treatment of Obsessive-Compulsive Disorder**

Total Number of Subjects Screened over the phone

N = 75

Subjects screened by phone with:

- Inclusion/Exclusion Criteria- DSM-5 criteria for OCD

Total Number of Subjects Scheduled for First Appointment

N = 55

17= did not arrive for first visit

10= called and told staff they were no longer interested in study

Total Number of Subjects Enrolled in Study (i.e. Signed Consent Forms)

N = 28

Total Number of Subjects Excluded after Signing Consent

n= 8

(deemed ineligible based on exclusion/inclusion criteria; or failed to attend baseline)

Screening Assessments:

**Investigator Administered**:

DSM-5 OCD criteria, YBOCS, HAM-A, HAM-D, Medical History

Total Number of Subjects Randomized

N = 20

Total Number of Subjects Assigned to Tolcapone for the Initial 2-week phase

N = 10

Total Number of Subjects

Assigned to Placebo for the Initial 2-week phase

N = 10

Assessments done every 2 weeks:

**Investigator Administered**:

YBOCS, HAM-A, HAM-D

Assessments done every 2 weeks:

**Investigator Administered**:

YBOCS, HAM-A, HAM-D

Discontinued after the Tolcapone Phase had ended

N = 1 (10.0%)

Discontinued After the Placebo Phase had ended

N = 1 (10.0%)

(n=x lost to follow-up)

**Total Number of Subjects Who Completed the Study**

**N =18 (90%)**

Total Number of Subjects Assigned Initially to Tolcapone who completed the entire study

N = 9 (90%)

Total Number of Subjects Assigned Initially to Placebo who completed the entire study

N = 9 (90%)

**YBOCS –**Yale Brown Obsessive Compulsive Scale; **HAM-A** – Hamilton Anxiety Rating Scale; **HAM-D** – Hamilton Depression Rating Scale;
